# Sometimes karyotype resolves the case!

**DOI:** 10.3389/fgene.2024.1371166

**Published:** 2024-02-28

**Authors:** Laura Rodríguez, Elena Barros, Jesica Skaarup Murray

**Affiliations:** ^1^ Genetic Laboratory AbaCid, HM Hospitales, Hospital Universitario HM Sanchinarro, Madrid, Spain; ^2^ EGOM (Equipo Médico de Ginecología y Obstetricia), HM Hospitales, Madrid, Spain

**Keywords:** chorionic villus biopsia, QF-PCR, array CGH, mosaicism, karyotype, amniocentesis

Currently, there is a general tendency to assume that cytogenetics is going to disappear and that it is going to be replaced by new molecular genetic technologies. It is evident that these new technologies have come to bring significance to genetics and genetic diagnosis, with all the advances that this entails for the benefit of patients (Hochstenbach et al., 2019). Patients and their families benefit since, if they fall into expert hands, the “diagnostic odyssey” from which many of them currently continue to suffer is greatly reduced (Chen et al., 2020). However, with the presentation of this case, we would like to vindicate the importance of cytogenetics, in particular karyotyping, to resolve some cases (Liehr T. 2021). In the present case, the study of the chorionic villus biopsy with quantitative fluorescence–polymerase chain reaction (QF-PCR), karyotype, and array-based comparative genomic hybridization (array CGH), at the beginning, did not resolve the case. Finally, the amniocentesis karyotype showed the alteration present in the fetus and clarified what had happened.

Chorionic villus sampling (CVS), or chorionic villus biopsy, is a prenatal test that involves taking a sample of tissue from the placenta (a structure in the uterus that provides blood and nutrients from the mother to the fetus), to perform a prenatal study with the advantage of prematurity of the results (usually done between weeks 10 and 12 of pregnancy). Chorionic villi are tiny projections of placental tissue that look like algae and generally contain the same genetic material as the fetus (Levy and Stosic, 2019). Nevertheless, the observation of multiple, chromosomally distinct cell lines in chorionic villus samples is not an unusual finding and occurs in 1 per 100 samples, which implies a risk to be taken into account in every CVS performed in hospitals (Schreck et al., 1990; Coorens et al., 2021; West and Everett, 2022).

A fetal nuchal translucency of 4.7 mm was detected in a fetus in the 12th week of pregnancy, and therefore, a CVS was performed. The sample was seeded and kept in an incubator at 37°C for 3 weeks for karyotyping. Meanwhile, DNA was extracted to perform QF-PCR and array CGH. QF-PCR was performed following the procedure of the commercial company Devyser Compact v3 (2016), which analyzes microsatellite markers of chromosomes 13, 18, 21, X, and Y. The result was reported as a female fetus with two chromosomes from each of the pairs analyzed (named as 13, 18, and 21) (maternal cell contamination was ruled out). It was observed that three microsatellite markers located in Xq26.1, Xq26.2, and Xq27.1 had a proportion of areas at the limit of normality (Figure 1A).

**FIGURE 1:**
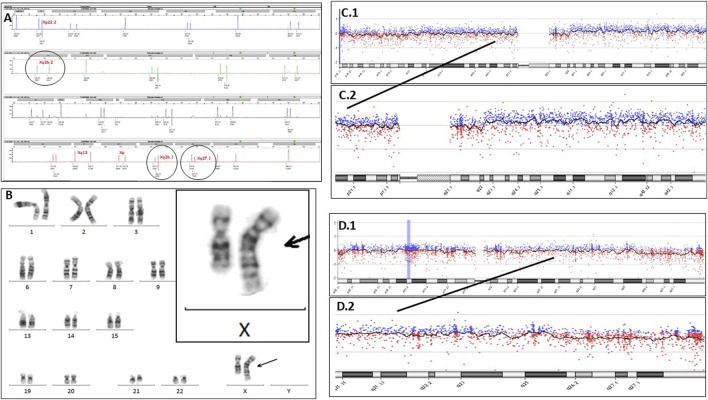
**(A)** Electropherogram of QF-PCR, using Devyser Compact v3, showing, in detail, the microsatellites of the X chromosome located in Xq26.1, Xq26.2, and Xq27.1. **(B)** Karyotyping of the amniotic fluid showing a chromosomal alteration between chromosomes 1 and X in 100% of the analyzed cells (46,XX, der (X)t (1; X) (q23.1; q25). **(C1)** Chromosome 1 array CGH image. **(C2)** Detail of the long arm (q) of chromosome 1, showing a very slight upward deviation, suggesting the presence of duplication in a mosaicism form. **(D1)** Chromosome X array CGH image. **(D2)** Long arm (q) of chromosome X, showing a very slight downward deviation, suggesting the presence of deletion in a mosaicism form.

Array CGH was performed following the usual procedure, and the sample showed a genomic pattern of female sex; no copy number change variations in a non-polymorphic nature were detected [array-CGH (ISCN 2020):arr (1-22,X)x2)]. For more information, the following can be observed: Labeling and hybridization of the sample with a commercial female reference DNA (Promega Biotech) on a KaryoNIM®prenatal array CGH platform and array CGH 60000 oligonucleotides focused on pathological prenatal syndromes (NIMGenetics^®^, Agilent Technologies). The hybridized platform was scanned with an Agilent scanner model G2565CA, using Agilent Scan Control 8.4.1 software (scanning resolution 3 μm), and analyzed with Agilent Cytogenomics 2.0 (NCBI37 genomic construct).

After 3 weeks, the cultured cells were harvested, and a normal female karyotype was obtained.

The pregnancy continued, and in week 16 of gestation, an omphalocele was diagnosed in the fetus sonographically; as a consequence, amniocentesis was decided to be performed. With this sample, the parents decided not to perform either QF-PCR or array CGH and accepted only a karyotype; so the sample was cultured in the incubator, and the parents waited 3 weeks for the result. After which, surprisingly, the karyotype showed an alteration in the long arm of one of the X chromosomes. When viewed in detail by experts, a banding pattern that coincided with the bands of the long arm of a chromosome 1 was revealed, showing a partial deletion of the long arm of an X chromosome together with a partial duplication of the long arm of chromosome 1 [46,XX, der (X)t (1; X) (q23.1; q25)](Figure 1B). This alteration was present in all the analyzed amniocytes (25 cells) and was confirmed as apparently “*de novo*”. It justified the malformations observed in the fetus and also suggested many more problems in the future baby. This is due to the fact that translocations involving the X chromosome have an added complexity since in females, one X chromosome is randomly inactivated, and in cases of unbalanced X-autosomal translocations, there is preferential silencing of the abnormal X chromosome (Strong et al., 2023) with the possible consequent inactivation of autosomal genes. With all the information exposed and a complex genetic counselling session being followed, the parents decided to terminate the pregnancy.

Following these findings, some listed questions arise to be resolved: **First,** what happened to QF-PCR of CVS? It should be noted that the three markers located in the terminal region of the long arm of the X chromosome were at the limit of normality, which showed a mosaic pattern confined to the placenta, which went unnoticed. **Second,** what happened to array CGH of the CVS? Now that we know, if we look in detail at the pattern of the specific array of chromosomes 1 and X, we can sense the alteration (Figures 1C1, C2, D1, D2), but it went completely unnoticed at the time. For more details, the array CGH detected below the limit of resolution, a possible deletion in the Xq25q28 cytobands (genomic coordinates chrX:126,563,609-154,908,612; 28.35 megabases, containing 1175 OMIM-listed genes), and, also below the resolution limit, a possible duplication in the cytobands 1q23.1q44 (genomic coordinates chr1:157103852_248903563; 91.8 metabases, containing 555 OMIM-listed genes). The signal intensity observed in both regions suggests that the duplication and deletion are in a mosaic state below 40%. **Third,** what happened to the CVS karyotype? Here, we have two possible answers: one is that the cells that grew were from the mother, and the mother’s karyotype was reported; another possible answer is that the normal cell line of the mosaic confined to the placenta grew; both possibilities resulted as a female normal karyotype, as reported.

Therefore, the karyotype performed on amniocytes revealed the baby’s real chromosome set and the presence of the chromosomal alteration present in 100% of the cells. This highlights the importance of karyotypes to understand the etiology of chromosomal rearrangements, which is essential for an appropriate follow-up and for ascertaining recurrence risks, as previously proposed by Hochstenbach et al., in 2021. Furthermore, with the presentation of this case, we claim the importance of a multidisciplinary approach when studying any case in the laboratory, in which each of the technologies contributes to different perspectives/results for a global vision of what happens in each of them, and most importantly, to be able to offer a correct genetic counselling to the family.

## References

[B1] Coorens T. H. H. Oliver T. R. W. Sanghvi R. Sovio U. Cook E. Vento-Tormo R. (2021). Inherent mosaicism and extensive mutation of human placentas. Nature 592, 80–85. 10.1038/s41586-021-03345-1 33692543 PMC7611644

[B2] Devyser Compact v3 (2016). Rapid prenatal diagnostics. Available at: https://www.bmd.be/wp-content/uploads/2017/05/Devyser_CompactV3_v2016-10-18_Web.pdf.

[B3] Hochstenbach R. Liehr T. Hastings R. J. (2021). Chromosomes in the genomic age. Preserving cytogenomic competence of diagnostic genome laboratories. Eur. J. Hum. Genet. 29, 541–552. 10.1038/s41431-020-00780-y 33311710 PMC8115145

[B4] Hochstenbach R. Van Binsbergen E. Schuring-Blom H. Buijs A. Ploos Van Amstel H. K. (2019). A survey of undetected, clinically relevant chromosome abnormalities when replacing postnatal karyotyping by Whole Genome Sequencing. Eur. J. Med. Genet. Sep. 62 (9), 103543. 10.1016/j.ejmg.2018.09.010 30248410

[B5] Levy B. Stosic M. (2019). Traditional prenatal diagnosis: past to present. Methods Mol. Biol. 1885, 3–22. 10.1007/978-1-4939-8889-1_1 30506187

[B6] Liehr T. (2021). About classical molecular genetics, cytogenetic and molecular cytogenetic data not considered by Genome Reference Consortium and thus not included in genome browsers like UCSC, Ensembl or NCBI. Mol. Cytogenet 14, 20. 10.1186/s13039-021-00540-7 33743766 PMC7981792

[B7] Schreck R. R. Falik-Borenstein Z. Hirata G. (1990). Chromosomal mosaicism in chorionic villus sampling. Clin. Perinatol. 17 (4), 867–888. 10.1016/s0095-5108(18)30550-5 2286032

[B8] Strong A. Callahan K. P. Guo R. Ron H. Zackaia E. H. (2023). X-Autosome translocations: X-inactivation and effect on phenotype. Clin. Dysmorphol. 30 (4), 186–188. 10.1097/MCD.0000000000000381 PMC9969345 34148988

[B9] West J. D. Everett C. A. (2022). Preimplantation chromosomal mosaics, chimaeras and confined placental mosaicism. Reprod. Fertil. 3 (2), R66–R90. 10.1530/RAF-21-0095 35514539 PMC9066951

[B10] Wu C. McMahon P. Lu C. (2020). Ending the Diagnostic Odyssey: is whole genome sequencing the answer? JAMA Pediatr. 174 (9), 821–822. 10.1001/jamapediatrics.2020.1522 32597967 PMC7928067

